# Diagnostic accuracy of ultrasound superb microvascular imaging for lymph nodes: A protocol for systematic review and meta-analysis

**DOI:** 10.1097/MD.0000000000022034

**Published:** 2020-09-04

**Authors:** Xuejiao Li, Cong Wang, Xiukun Hou, Ye Tao

**Affiliations:** Ultrasound department of the First Affiliated Hospital of Dalian Medical University, China.

**Keywords:** lymph node, meta-analysis, superb microvascular imaging

## Abstract

**Background:**

As a novel ultrasound technique, superb microvascular imaging can quickly, simply and noninvasively study the microvascular distribution in the tumor and evaluate the microvascular perfusion. Studies suggested that superb microvascular imaging is helpful for the differentiation between benign and malignant lymph nodes. However, the results of these studies have been contradictory. Therefore, the present meta-analysis aimed at determining the accuracy of superb microvascular imaging in the differential diagnosis between benign and malignant lymph nodes.

**Methods:**

We will search PubMed, Web of Science, Cochrane Library, and Chinese biomedical databases from their inceptions to the July 30, 2020, without language restrictions. Two authors will independently carry out searching literature records, scanning titles and abstracts, full texts, collecting data, and assessing risk of bias. Review Manager 5.2 and Stata14.0 software will be used for data analysis.

**Results:**

This systematic review will determine the accuracy of superb microvascular imaging in the differential diagnosis between benign and malignant lymph nodes.

**Conclusion:**

Its findings will provide helpful evidence for the accuracy of superb microvascular imaging in the differential diagnosis between benign and malignant lymph nodes.

**Systematic review registration:**

INPLASY202070133.

## 1 Introduction

Lymph nodes belong to important peripheral immune organ, which participates in the immune response of the body.^[1]^ Lymphadenopathy is very common in clinic.^[2]^ The etiology mainly includes local or systemic inflammatory reaction, metastasis of malignant tumor to lymph node, lymphoma, and so on.^[3]^ High frequency ultrasound can not only show the size, shape, and internal echo of lymph nodes, but also show the blood flow distribution.^[4]^ As a novel ultrasound technique, superb microvascular imaging can quickly, simply and noninvasively study the microvascular distribution in the tumor and evaluate the microvascular perfusion.^[5]^ The SMI adopts a multidimensional filter to eliminate only the clutter and to preserve low-velocity flow signals, whereas conventional Doppler systems use a single-dimension filter and, accordingly, can exhibit a loss of low-velocity flow signals that overlap with clutter.^[6]^ Studies suggested that superb microvascular imaging is helpful for the differentiation between benign and malignant lymph nodes.^[7–10]^ However, the results of these studies have been contradictory. Therefore, the present meta-analysis aimed at determining the accuracy of superb microvascular imaging in the differential diagnosis between benign and malignant lymph nodes.

## 2 Materials and methods

This study was conducted in accordance with the PRISMA (Preferred Reporting Items for Systematic Reviews and Meta-Analyses) guidelines and the protocol was registered in the INPLASY (INPLASY202070133).

### 2.1 Eligibility criteria

#### 2.1.1 Type of study

This study will only include high quality clinical cohort or case control studies.

#### 2.1.2 Type of patients

The patients should be those who had undergone Lymphadenopathy.

#### 2.1.3 Intervention and comparison

This study compare SMI with pathology for diagnosing lymph nodes.

#### 2.1.4 Type of outcomes

The primary outcomes include sensitivity, specificity, positive and negative likelihood ratio, diagnostic odds ratio, and the area under the curve of the summary receiver operating characteristic.

### 2.2 Search methods

PubMed, Web of Science, Cochrane Library, and Chinese biomedical databases will be searched from their inceptions to the July 31, 2020, without language restrictions. The search strategy for PubMed is shown in Table 1. Other online databases will be used in the same strategy.

**Table 1:**
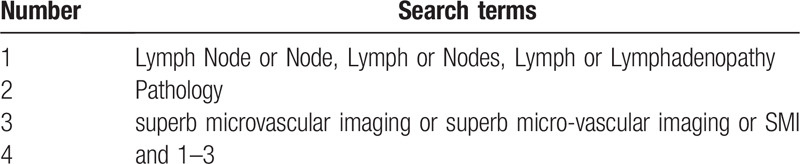
Search strategy sample of pubmed.

### 2.3 Data extraction and quality assessment

Two authors will independently select the trials according to the inclusion criteria, and import into Endnote X^9^. Then remove duplicated or ineligible studies. Screen the titles, abstracts, and full texts of all literature to identify eligible studies. All essential data will be extracted using previously created data collection sheet by 2 independent authors. Discrepancies in data collection between 2 authors will be settled down through discussion with the help of another author. The following data will be extracted from each included research: the first authors surname, publication year, language of publication, study design, sample size, number of lesions, source of the subjects, instrument, “gold standard,” and diagnostic accuracy. The true positives, true negatives, false positives, and false negatives in the fourfold (2 × 2) tables were also collected. Methodological quality was independently assessed by 2 researchers based on the quality assessment of studies of diagnostic accuracy studies (QUADAS) tool.^[11]^ The QUADAS criteria included 14 assessment items. Each of these items was scored as “yes” (2), “no” (0), or “unclear”(1). The QUADAS score ranged from 0 to 28, and a score ≥22 indicated good quality. Any disagreements between 2 investigators will be solved through discussion or consultation by a 3rd investigator.

### 2.4 Statistical analysis

The STATA version 14.0 (Stata Corp, College Station, TX, USA) and Meta-Disc version 1.4 (Universidad Complutense, Madrid, Spain) softwares were used for meta-analysis. We calculated the pooled summary statistics for sensitivity, specificity, positive and negative likelihood ratio, and diagnostic odds ratio with their 95% confidence intervals. The summary receiver operating characteristic curve and corresponding area under the curve were obtained. The threshold effect was assessed using Spearman correlation coefficients. The Cochrans Q-statistic and *I* test were used to evaluate potential heterogeneity between studies. If significant heterogeneity was detected (*Q* test *P* < .05 or *I* test > 50%), a random effects model or fixed effects model was used. We also performed sub group and meta-regression analyses to investigate potential sources of heterogeneity. To evaluate the influence of single studies on the overall estimate, a sensitivity analysis was performed. We conducted Beggs funnel plots and Eggers linear regression tests to investigate publication bias.

### 2.5 Ethics and dissemination

We will not obtain ethic documents because this study will be conducted based on the data of published literature. We expect to publish this study on a peer-reviewed journal.

## 3 Discussion

The vascular morphology and distribution of lymph nodes are closely related to their nature, which can be used as an important supplementary sign in their differential diagnosis.^[12,13]^ SMI can clearly and completely show the shape and distribution of vascular network of lymph node without injection of contrast agent.^[14]^ Studies suggested that superb microvascular imaging is helpful for the differentiation between benign and malignant lymph nodes. However, the results of these studies have been contradictory. To gain clarity, in this study, we will perform a systematic review to summarize high-quality studies and to provide evidence on the evidence-based medical support for clinical practice.

## Author contributions

**Conceptualization:** Ye Tao and Xiukun Hou.

**Data curation:** Cong Wang and Xuejiao Li.

**Methodology:** Cong Wang and Xuejiao Li.

**Writing – original draft:** Cong Wang and Xuejiao Li.

**Writing – review & editing:** Cong Wang, Xuejiao Li, and Xiukun Hou.
